# LncRNAs act as prognostic and diagnostic biomarkers in renal cell carcinoma: a systematic review and meta-analysis

**DOI:** 10.18632/oncotarget.11101

**Published:** 2016-08-05

**Authors:** Jianwen Chen, Yalei Chen, Liangyou Gu, Xintao Li, Yu Gao, Xiangjun Lyu, Luyao Chen, Guoxiong Luo, Lei Wang, Yongpeng Xie, Junyao Duan, Cheng Peng, Xin Ma

**Affiliations:** ^1^ Department of Urology/State Key Laboratory of Kidney Diseases, Chinese People's Liberation Army General Hospital, Beijing, China; ^2^ Department of Cardiology, Beijing Anzhen Hospital affiliated to Capital Medical University, Beijing, China

**Keywords:** lncRNA, renal cell carcinoma, clinicopathology, diagnosis, prognosis

## Abstract

We conducted a systematic review and meta-analysis to investigate the clinical values, including clinicopathology, prognosis, and diagnosis of different long non-coding RNAs (lncRNAs) in renal cell carcinoma (RCC). A total of 14 eligible studies, including 10 on clinicopathological features, 11 on prognosis, and 3 on diagnosis were identified. Results revealed that metastasis-associated lung adenocarcinoma transcript 1(MALAT1) expression was associated with tumor stage (odds ratio [OR], 3.46; 95% confidence interval [CI], 1.63-7.36; *p*=0.001). The high expression of MALAT1 could be considered a biomarker of the early detection of lymph node metastasis and predictor of poor survival in RCC patients, who likely manifested short overall survival (OS; hazard ratio [HR], 2.97; 95% CI, 1.68-5.28; *p*<0.001). For diagnostic value, the pooled result showed that lncRNA maintained a sensitivity of 0.89 and specificity of 0.91 in RCC diagnosis, The area under the curve of 0.94 (95% CI, 0.92-0.96) for lncRNA in RCC diagnosis also indicated a significant advantage over other biomarkers. Our systematic review and meta-analysis demonstrated that lncRNAs could be considered biomarkers to detect lymph node metastasis and distant metastasis in early stages. LncRNAs could function as potential prognostic markers in RCC. LncRNAs could also display high accuracy for RCC diagnosis.

## INTRODUCTION

It is estimated that about 66,800 Chinese will suffer from kidney cancer in 2015, and about 23,400 Chinese will die from this cancer [1]. The incidence of renal cell carcinoma has rapidly increased [2]. RCC is the most common form of kidney cancer in adults, and clear cell RCC (ccRCC) is the most common subtype; patients with advanced RCC have a 5 year survival rate of < 30% [3]. Surgery is the gold standard for localized RCC; however, this strategy provides limited benefits for patients with locally advanced or metastatic RCC; Metastatic RCC is also resistant to chemotherapy and radiotherapy; as such, new therapeutic targets should be developed. In RCC research, several genetic biomarkers, including mRNAs, such as HIF1α [4], Von Hippel-Lindau gene(VHL) [4], NOTCH1 [5], S100A6 [6], and E2F1 [7], and microRNAs (miRs), such as miR-21 [8], miR-221 [9] and miR-30a [10], have been used. Long non-coding RNAs (lncRNAs) have also been extensively investigated because of their clinical usefulness and biological properties in diagnosis, prognosis, and treatment.

LncRNAs are a class of RNA with transcripts longer than 200 nucleotides and lack functional open reading frames [11]. These RNAs actively function in various cell biological processes, such as cellular differentiation, proliferation, DNA damage response, and chromosomal imprinting [12]. An increasing number of studies have shown that lncRNAs were closely related to many human diseases, including cancer [13, 14]. Their expression profiling in various cancer types have been widely examined, and many of these lncRNAs were correlated with cancer diagnosis and prognosis. For example, lncRNA-XIST, a product of the X-inactive specific tran­script gene, and lncRNA HIF 1 alpha-antisense RNA 1 (HIF1a-AS1) are up-regulated in non-small cell lung cancer (NSCLC), and can be used as a diagnostic biomarker for NSCLC screening [15]. Urothelial carcinoma-associated-1(UCA1) is highly expressed in the plasma of Gastric Cancer (GC) patients, and can be a promising noninvasive diagnostic biomarker for GC [16]. lncRNA UCA1 is also identified as sensitive diagnostic markers for bladder cancer [17]. lncRNA-ATB, a TGF-β-activated lncRNA, is significantly up-regulated in hepatocellular carcinoma metastases and associated with poor prognosis [18]. HOX transcript antisense intergenic RNA (HOTAIR) is also correlated with the tumor stage and poor prognosis of non-small-cell lung cancer; the down-regulation of HOTAIR inhibits the invasion and metastasis of non-small-cell lung cancer cells through the down-regulation of HOXA5 [19]. Recent studies have shown that lncRNAs are also potential diagnostic and prognostic biomarkers of RCC; these finding suggest that these RNAs can be developed as biomarkers to guide therapeutic decisions [20-22].

However, single study may be inaccurate and insufficient because of limitations related to sample size and research programs. As such, studies should be systematically analyzed to determine the potential clinical values of lncRNAs in RCC. Thus far, meta-analysis has yet to be performed, although some reviews have been conducted regarding the evaluation of the clinical values of different lncRNAs in RCC. Likewise, the clinical values of lncRNAs have been rarely analyzed. Therefore, we systematically reviewed studies that have identified the relationship between lncRNA expression and clinical outcomes in RCC. We also included these studies in our meta-analysis if extracted data could be merged. We mainly discussed our findings in terms of the following aspects: clinicopathological features, diagnosis, and prognosis.

## RESULTS

### Study characteristics

A total of 464 records were retrieved from PubMed, Embase and Web of science. A total of 173 duplicate reports were excluded. After the titles were reviewed, 181 records were excluded. After the abstracts were screened, 73 records were excluded. Subsequently, the 37 remaining full-text articles were assessed, and 23 studies, including 10 without clinical data, 1 with less than 30 sample numbers, 2 with description on genetic variation, 2 duplicate articles, 2 with discussion on other diseases, 2 with discussion on lncRNA-methylation, and 4 microarray articles, were further excluded on the basis of the exclusion criteria. A total of 14 studies, including 10 on clinicopathological features, 11 on prognosis, and 3 on diagnosis, were eligible for the final analysis. All of the selected studies were nonrandomized. A flow diagram of the study selection process is shown in Figure 1.

**Figure:**
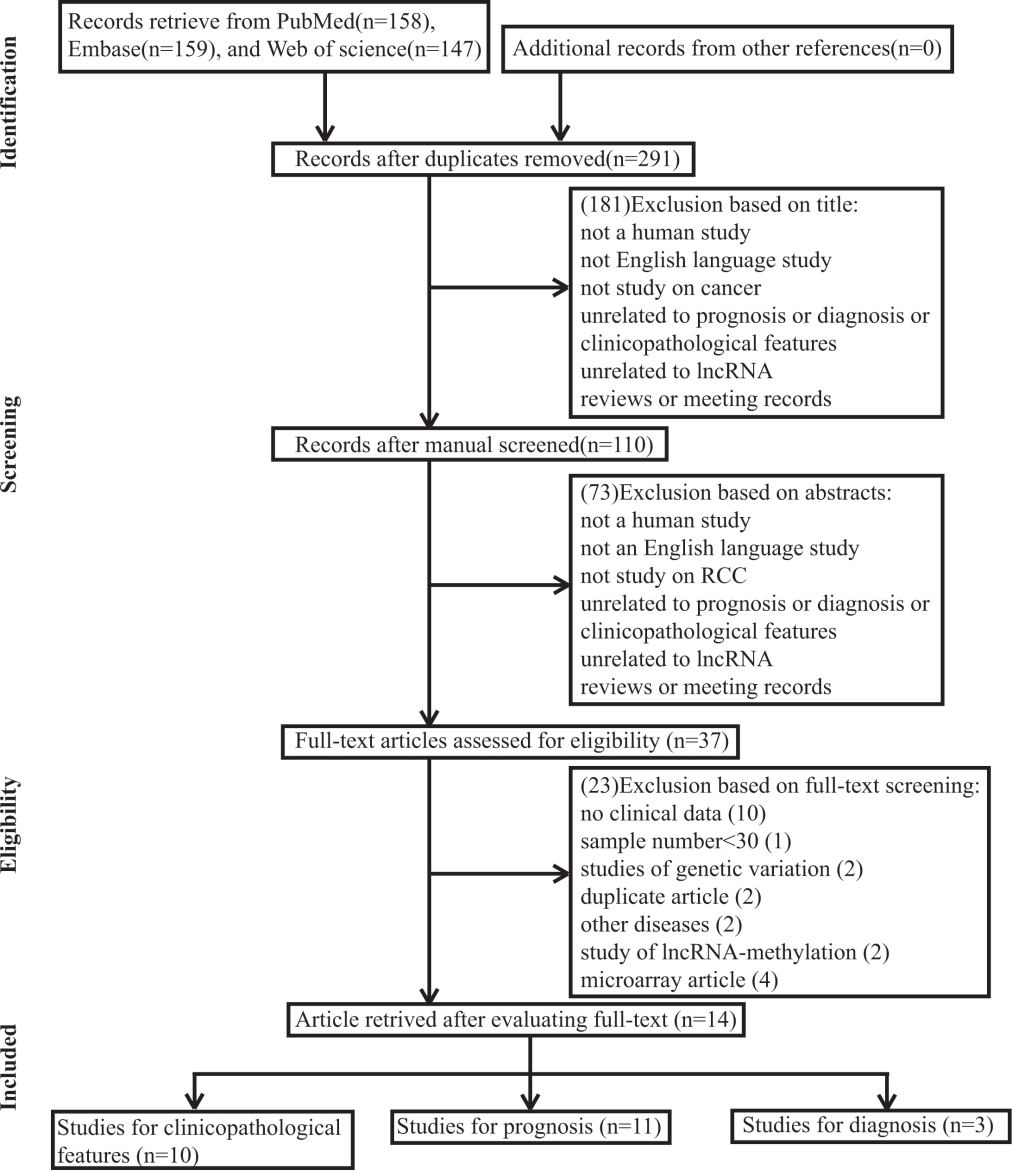
Flow diagram of study selection process

### Clinicopathological features

**Figure 2:**
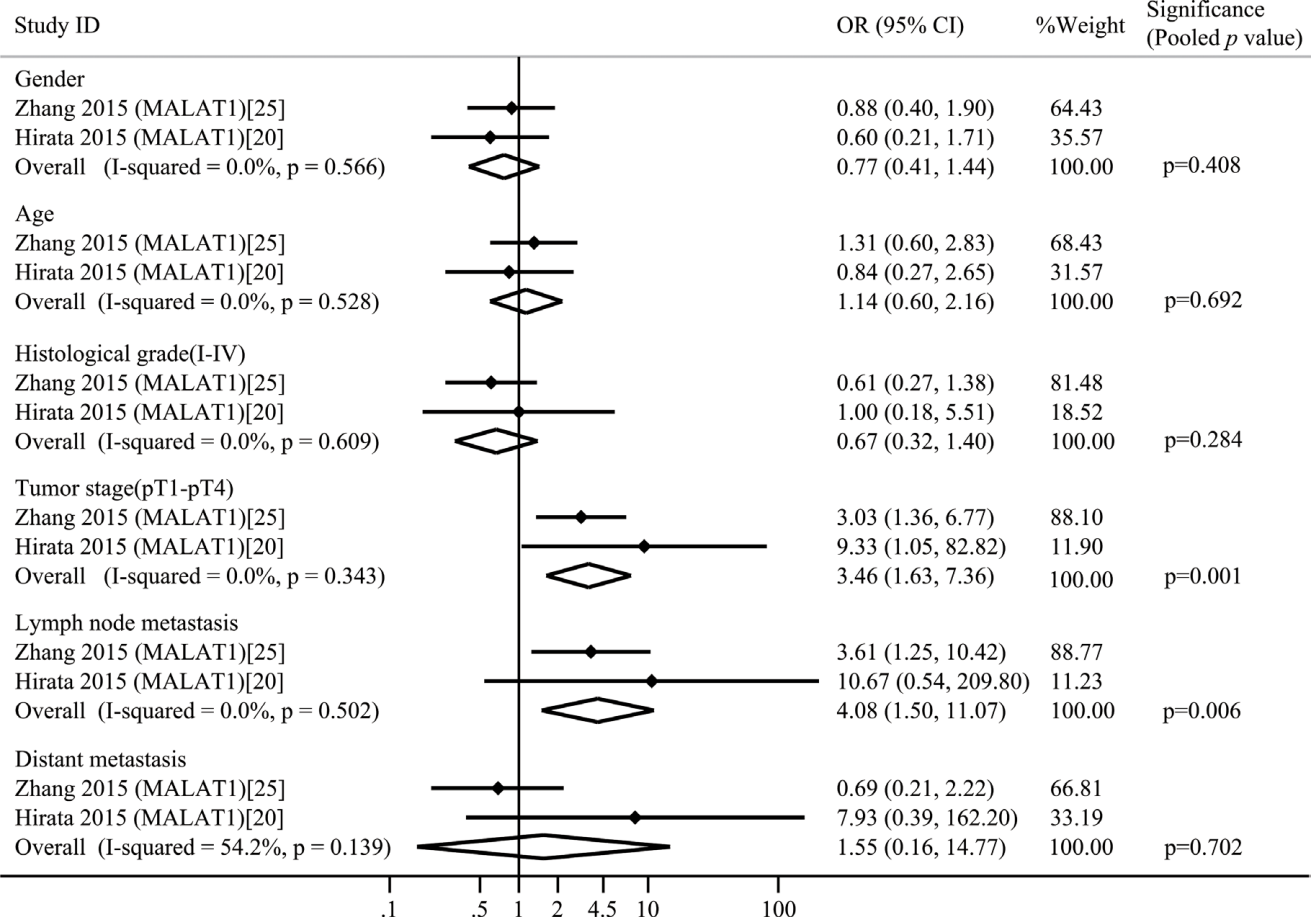
Forest plots of studies evaluating odds ratios (ORs) of up-regulated MALAT1 expression and the clinicopathology of RCC patients

A total of 9 lncRNAs were described in the 10 included studies on clinicopathological features. renal cell carcinoma related transcript-1(RCCRT1) [23], protein sprouty homolog 4 intronic transcript-1 (SPRY4-IT1) [24], H19 [21], metastasis-associated lung adenocarcinoma transcript 1(MALAT1) [20, 25], and lncRNA activated by TGF-β (ATB) [22] were up-regulated, and Cell adhesion molecule 1 anti-sense transcript-1 (CADM1-AS1) [26], neuroblastoma associat­ed transcript-1 (NBAT-1) [27], lnc-ZNF180-2 [28], and NONHSAT123350 [29] were down-regulated. None of these studies reported that lncRNAs were significantly associated with gender and age of patients. Ellinger [28] revealed that clinicopathological characteristics were not significantly related to the expression levels of lnc-ZNF180-2. Two studies claimed that up-regulated RCCRT1 [23] and MALAT1 [25] were significantly related to tumor size. Five studies demonstrated that lncRNAs were significantly correlated with histological-grade RCC, while the most of the studies reported that lncRNAs were significantly correlated with tumor stage (Table 1). MALAT1 was detected in two studies. We constructed two-by-two tables to calculate the odds ratio (OR) and *p* value of these two studies by extracting the raw patient number. We then combined these two studies with a total of 6 groups (Figure 2). Moderate heterogeneity was observed in one group (distant metastasis, I^2^ = 54.2%); therefore, a random effects model was used in this study; for other cases, a fixed effects model was utilized. After combining these two studies, we found that the statistical significance of one group changed. Before combination was performed, Hirata *et al.* [20] reported that up-regulated MALAT1 was not significantly correlated with lymph node metastasis because OR was 10.67 (0.54, 209.80). However, the pooled OR and *p* values indicated that this correlation was indeed significant. The other comparisons of MALAT1 did not indicate significant changes.

**Table 1:** Summary of the comparison for the p values of the association between lncRNAs and clinicopathological features

Studies	LncRNAs	population	Case number	Cut-off value	Gender	Age	Tumor size (cm)	Histological grade(I-IV)	Tumor stage(pT1-pT4)	Lymph node metastasis	Distant metastasis	Expression
Song 2014	RCCRT1[23]	Chinese	40	fold-change	0.085	0.728	0.046	0.017	0.022	0.008	0.003	up-regulation
Zhang 2014	SPRY4-IT1[24]	Chinese	98	mean	0.888	0.648	0.878	0.002	<0.001	0.001	0.003	up-regulation
Yao 2014	CADM1-AS1[26]	Chinese	64	median	0.611	0.606	0.578	0.133	0.039	NA	NA	down-regulation
Xue 2015	NBAT-1[27]	Chinese	98	median	0.685	0.068	0.835	0.006	<0.001	0.021	NA	down-regulation
Wang 2015	H19[21]	Chinese	92	fold-change	0.993	0.463	0.087	<0.001	0.002	0.001	0.01	up-regulation
Ellinger2015	lnc-ZNF180-2[28]	German	91	median	>0.7	>0.7	>0.7	>0.7	>0.7	>0.7	>0.7	down-regulation
Zhang 2015	MALAT1[25]	Chinese	106	mean	0.744	0.495	<0.001	0.235	0.006	0.014	0.534	up-regulation
Hirata 2015	MALAT1[20]	Japanese	50	median	0.967	0.609	NA	0.217	0.001	0.003	0.077	up-regulation
Liu 2016	NONHSAT123350[29]	Chinese	90	risk quotient	NA	0.4*	0.21*	NA	0.3*	NA	−0.92*	down-regulation
Xiong 2016	lncRNA-ATB[22]	Chinese	74	median	0.450	0.363	NA	0.011	0.030	0.013	0.015	up-regulation

NA=not available;

* Correlation coefficient value, RCCRT1=renal cell carcinoma related transcript-1, SPRY4-IT1= protein sprouty homolog 4 intronic transcript-1

CADM1-AS1= Cell adhesion molecule 1 anti-sense transcript-1, NBAT-1=neuroblastoma associat­ed transcript-1, MALAT1= metastasis-associated lung adenocarcinoma transcript 1, lncRNA-ATB=a novel lncRNA activated by TGF-β

### Prognosis

**Figure 3:**
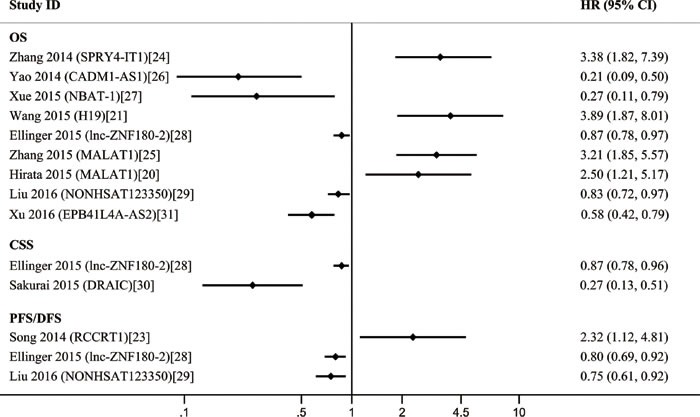
A display of Hazard ratios (HRs) of lncRNAs in RCC patients The point estimate is bounded by a 95% confidence interval (CI), and the perpendicular line represents no increased risk for the outcome. OS = overall survival; CSS = cancer specific survival; PFS = prognostic free survival; DFS = disease free survival.

**Figure 4:**
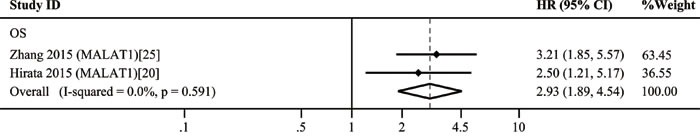
Forest plots of studies evaluating hazard ratios of up-regulated MALAT1 expression and the overall survival (OS) of RCC patients

The 11 included studies were retrospective and published over the recent two years. Quantitative real-time polymerase chain reaction (qRT-PCR) was performed to evaluate the lncRNA expression in 1,510 tissue samples. Ten different lncRNAs were associated with the prognosis of patients with RCC. The tumor type of patient in ten studies was ccRCC. The characteristics of these 11 eligible studies are presented in Table 2. The increased expressions of RCCRT1 [23], SPRY4-IT1 [24], H19 [21], and MALAT1 [20, 25] were associated with poor prognosis; likewise, the decreased expressions of CADM1-AS1 [26], NBAT-1 [27], lnc-ZNF180-2 [28], NONHSAT123350 [29], down-regulated RNA in androgen independent cells (DRAIC) [30], and EPB41L4A-AS2 [31] (Figure 3) were related to poor prognosis. The sample size of these studies was more than 40; of these studies, the study of Xu et al. [31] included the largest case number of 448. H19 [21] yielded the highest hazard ratio (HR) of 3.89; by contrast, CADM1-AS1 [26] exhibited the lowest HR of 0.21. A lncRNA was considered to be a weak prognostic factor with HR between 0.67 and 1.5 [32]. At this point of view, although the correlations among all of these lncRNAs with prognosis were statistically significant, lnc-ZNF180-2 [28] and NONHSAT123350 [29] were insufficiently strong. All of these ten lncRNAs except MALAT1 were investigated in single research; MALAT1 was performed in two studies. We then conducted a meta-analysis on the relationship of MALAT1 expression and the overall survival (OS) of patients with RCC; we found that heterogeneity was not significant (I^2^ = 0.0%, *p* = 0.591). Therefore, the fixed effects model was applied. The model revealed that high MALAT1 expression could predict short OS (HR, 2.93; 95% confidence interval [CI] 1.89-4.54; *p* < 0.001; Figure 4). No conclusive graph could be generated because of the small size of this study. Therefore, we did not evaluate publication bias.

**Table 2:** Summary of lncRNAs used as prognostic biomarkers of RCC

Study	LncRNA name	Region	Study design	Tumor type	Tumor stage	Detected sample	Assay methods	Cut-off method	Case number		Survival analysis	HR availability	Follow-up month
									High level	Low level			
Song 2014	RCCRT1[23]	China	R	ccRCC	pT1-pT4	FT	qRT-PCR	fold change	24	16	PFS	Indirectly	14(8-22)
Zhang 2014	SPRY4-IT1[24]	China	R	ccRCC	pT1-pT4	FT	qRT-PCR	mean	52	46	OS	Directly	35(0-60)
Yao 2014	CADM1-AS1[26]	China	R	ccRCC	pT1-pT4	FT	qRT-PCR	median	32	32	OS	Directly	~80
Xue 2015	NBAT-1[27]	China	R	ccRCC	pT1-pT4	FT	qRT-PCR	median	49	49	OS	Directly	35(0-60)
Wang 2015	H19[21]	China	R	ccRCC	pT1-pT4	FT	qRT-PCR	fold-change	42	50	OS	Directly	~60
Ellinger 2015	lnc-ZNF180-2[28]	Germany	R	ccRCC	pT1-pT4	FT	qRT-PCR	median	46	45	PFS, CSS, OS	Directly	144
Zhang 2015	MALAT1[25]	China	R	ccRCC	I-IV	FT	qRT-PCR	mean	46	60	OS	Indirectly	~60
Hirata 2015	MALAT1[20]	Japan	R	ccRCC	pT1-pT4	FT	qRT-PCR	median	25	25	OS	Indirectly	47
Sakurai 2015	DRAIC[30]	America	R	ccRCC	NA	FT	qRT-PCR	Z-score	75	258	DFS	Indirectly	NA
Liu 2016	NONHSAT123350[29]	China	R	ccRCC	pT1-pT4	FT	qRT-PCR	RQV	32	58	DFS, OS	Indirectly	32(3-60)
Xu 2016	EPB41L4A-AS2[31]	NA	R	RCC	I-IV	FT	qRT-PCR	median	224	224	OS	Indirectly	~108

LncRNA=long non-coding RNA; R=Retrospective; ccRCC= clear cell renal cell carcinoma; RCC= renal cell carcinoma; FT=frozen tissue; qRT-PCR=quantities reverse transcription polymerase chain reaction; RQV=risk quotient value; PFS=prognostic free survival; OS=overall survival; CSS=cancer specific survival; DFS=disease free survival; HR = hazard ratio; NA=not available, DRAIC=downregulated RNA in androgen independent cells

### Diagnosis

**Figure 5:**
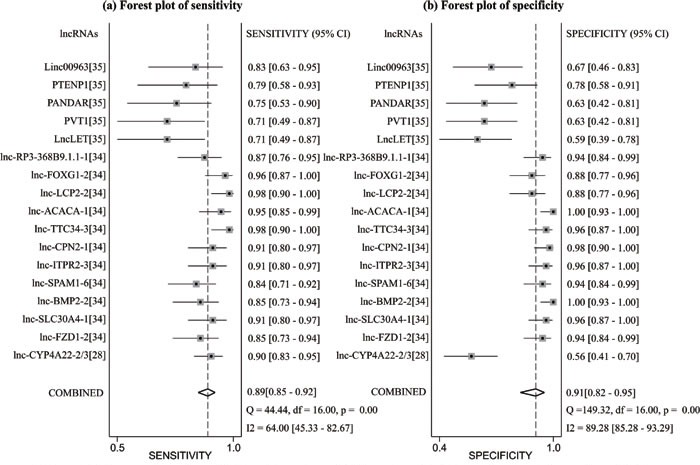
Forest plot of sensitivity a. and specificity b. of lncRNAs for the diagnosis of RCC

**Figure 6:**
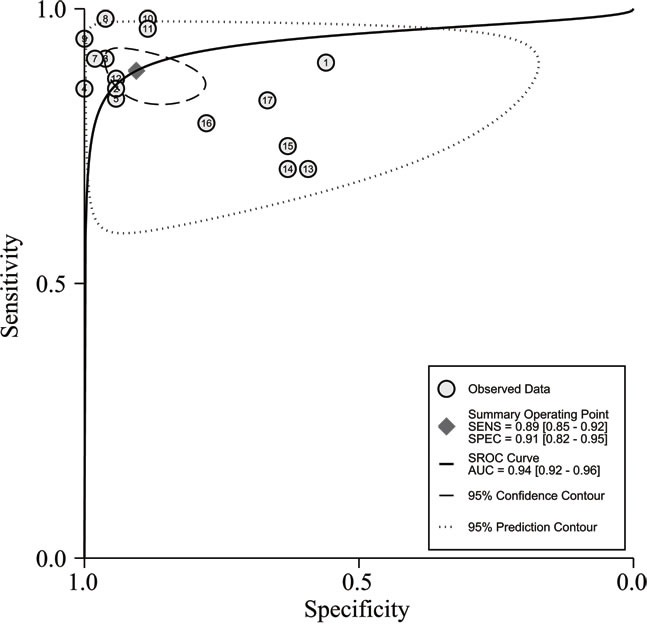
The summary receiver operator characteristic (SROC) curve based on all lncRNAs

In the diagnosis category, the main characteristics and Quality Assessment of Diagnostic Accuracy Studies-2 (QUADAS-2) [33] scores of each study are presented in Table 3. Three studies [28, 34, 35] containing 17 lncRNAs provided complete diagnostic data. Of these lncRNAs, 9 were down-regulated and 8 were up-regulated. LncRNA expression was detected through qRT-PCR in these studies. A total of 171 patients with ccRCC and 129 controls comprised patients with other diseases and healthy individuals. Two of the included studies used kidney tissues as specimens and one study was based on serum. All of these studies were published in 2015 and 2016. All included studies satisfied at least four of the seven items in QUADAS-2; this indicated that the overall quality of the included studies was generally good. Figure 4 presents the forest plots of sensitivity and specificity for the 17 lncRNAs. Significant heterogeneity between these studies was observed in sensitivity and specificity data (I^2^ = 64.00% and I^2^ = 89.28%, respectively). Therefore, the random effects model was used in this meta-analysis to calculate the pooled diagnostic parameters of the included studies. The pooled estimates of lncRNAs for the diagnosis of RCC were as follows: sensitivity(SEN), 0.89(95 % CI, 0.85-0.92); specificity(SPE), 0.91(95 % CI, 0.82-0.95); positive likelihood ratio(PLR), 9.4 (95 % CI, 4.8-18.4); negative likelihood ratio(NLR), 0.12 (95 % CI, 0.09-0.18); and overall diagnostic odds ratio (DOR), 75 (95 % CI, 29-193). Figure 5 displays the corresponding summary receiver operator characteristic (SROC) curve with an area under the curve (AUC) of 0.94 (95 % CI, 0.92 - 0.96). Thus the diagnostic accuracy of lncRNAs is relatively high. We also conducted meta-regression and subgroup analysis (Figure 6) on the bias of ethnicity, lncRNA expression level, and detected sample types. We found that detection process and ethnicity unlikely affect the diagnostic accuracy for RCC. By contrast, the relative expression of lncRNAs significantly influenced sensitivity. Hence, the down-regulation of lncRNA expression may exhibits a higher sensitivity in the diagnosis of RCC.

**Table 3:** Summary of lncRNAs used as diagnostic biomarkers of RCC

First author	Publish year	Country	Ethnicity	LncRNAs	Expression	SE(%)	SP(%)	AUC	Sample size		Mean age(yr)		Detected sample	QUADAS
									Cases Controls		Cases Controls			
Ellinger[28]	2015	Germany	Caucasian	lnc-CYP4A22-2/3	down-regulation	90.0	55.9	0.790	102	50	66	64.9	Frozen tissue	4
Blondeau[34]	2015	Germany	Caucasian	lnc-FZD1-2	up-regulation	85.5	94.2	0.931	55	52	62.9	62.1	Frozen tissue	5
Blondeau[34]	2015	Germany	Caucasian	lnc-SLC30A4-1	up-regulation	90.9	96.2	0.942	55	52	62.9	62.1	Frozen tissue	5
Blondeau[34]	2015	Germany	Caucasian	lnc-BMP2-2	up-regulation	85.5	100.0	0.912	55	52	62.9	62.1	Frozen tissue	5
Blondeau[34]	2015	Germany	Caucasian	lnc-SPAM1-6	up-regulation	83.6	94.2	0.900	55	52	62.9	62.1	Frozen tissue	5
Blondeau[34]	2015	Germany	Caucasian	lnc-ITPR2-3	up-regulation	90.9	96.2	0.941	55	52	62.9	62.1	Frozen tissue	5
Blondeau[34]	2015	Germany	Caucasian	lnc-CPN2-1	up-regulation	90.9	98.1	0.942	55	52	62.9	62.1	Frozen tissue	5
Blondeau[34]	2015	Germany	Caucasian	lnc-TTC34-3	down-regulation	98.1	96.4	0.990	55	52	62.9	62.1	Frozen tissue	5
Blondeau[34]	2015	Germany	Caucasian	lnc-ACACA-1	down-regulation	94.2	100.0	0.966	55	52	62.9	62.1	Frozen tissue	5
Blondeau[34]	2015	Germany	Caucasian	lnc-LCP2-2	down-regulation	98.1	89.1	0.955	55	52	62.9	62.1	Frozen tissue	5
Blondeau[34]	2015	Germany	Caucasian	lnc-FOXG1-2	down-regulation	96.2	89.1	0.954	55	52	62.9	62.1	Frozen tissue	5
Blondeau[34]	2015	Germany	Caucasian	lnc-RP3-368B9.1.1-1	down-regulation	86.5	94.5	0.938	55	52	62.9	62.1	Frozen tissue	5
Wu[35]	2016	China	Asian	LncLET	down-regulation	70.8	59.3	0.741	24	27	NA	NA	serum	6
Wu[35]	2016	China	Asian	PVT1	up-regulation	70.8	63.0	0.733	24	27	NA	NA	serum	6
Wu[35]	2016	China	Asian	PANDAR	down-regulation	75.0	63.0	0.738	24	27	NA	NA	serum	6
Wu[35]	2016	China	Asian	PTENP1	down-regulation	79.2	77.8	0.840	24	27	NA	NA	serum	6
Wu[35]	2016	China	Asian	Linc00963	up-regulation	83.3	66.7	0.812	24	27	NA	NA	serum	6

LncRNA=long non-coding RNA; RCC= renal cell carcinoma; SE= sensitivity; SP= specificity; AUC= area under the curve; NA=not available; QUADAS=quality assessment of diagnostic accuracy studies

**Figure 7:**
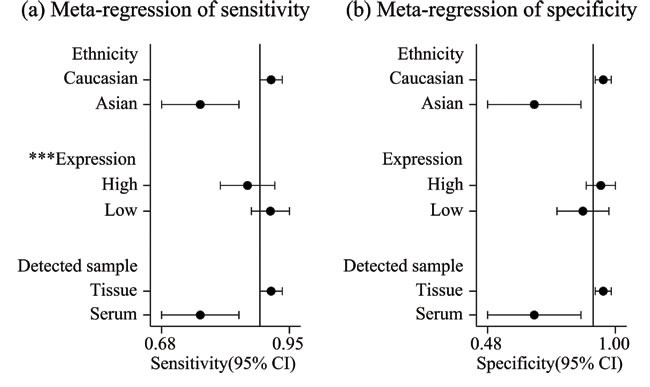
Univariate meta-regression and subgroup analysis for sensitivity a. and specificity b. of lncRNAs for the diagnosis of RCC(* *p* < 0.05, **p < 0.01, ***p < 0.001)

## DISCUSSION

Over the past decade, there were a lot of researches studying on lncRNAs. Increasing evidence showed that aberrant expression of lncRNAs was associated with clinical outcomes for cancer patients. LncRNAs were also closely related to cancer, they were considered eminent players in cancer biology. The up-regulation or down-regulation of many lncRNAs contributed to oncogenesis by affecting many cellular processes [36]. In order to find some biomarkers for RCC, we conducted this comprehensive systematic review and meta-analysis of the current literature. The present meta-analysis is the first to systematically analyze the association between lncRNA expression and clinical features of RCC, a total of 14 studies were included.

In the classification of clinicopathological features, dysregulated RCCRT1 [23], SPRY4-IT1 [24], H19 [21], NONHSAT123350 [29], and ATB [22] could potentially be exploited as novel biomarkers to detect distant metastasis in patients with RCC in early stages. Furthermore, RCCRT1 [23], SPRY4-IT1 [24], NBAT-1 [27], MALAT1 [25], and ATB [22] might be used as biomarkers of lymph node metastasis. Although these lncRNAs were associated with the clinicopathological features of patients with RCC; most of these lncRNAs were detected by single study; among these lncRNAs, MALAT1 was the most investigated and reported by two studies; this lncRNA was significantly associated with the tumor stage and lymph node metastasis of patients with RCC after we pooled OR and *p*-value. However, this conclusion may be insufficiently persuasive because these two studies used different cutoff values to distinguish high and low expression levels. Further studies should be performed because of the limitations of the included studies to verify these conclusions.

Regarding the prognostic value, the increased expression of four lncRNAs was associated with poor prognosis, as was the decreased expression of six lncRNAs. Although these lncRNAs were associated with the prognosis of patients with RCC, only MALAT1 was reported by two studies. We then conducted a meta-analysis on the relationship of MALAT1 expression and the OS of patients with RCC. The results revealed that a high MALAT1 expression predicted poor survival among patients with RCC; as such, these patients likely exhibited a short OS. However, this conclusion may be insufficiently persuasive because of the small size of this study. Thus, further research should be conducted to verify this conclusion.

For diagnostic value, the pooled result showed that lncRNA maintained high sensitivity and specificity in RCC diagnosis. PLR and NLR were applied to judge the clinical applicability of lncRNA for diagnosis, and the PLR > 10 and NLR < 0.1 represent a high diagnostic accuracy [32]. The pooled PLR and NLR obtained in our study showed a satisfactory diagnostic accuracy. The AUC of 0.94 for lncRNA in RCC diagnosis also indicated a significant advantage over other biomarkers. Considering that significant heterogeneity was observed in sensitivity and specificity data, we then performed a subgroup analysis on ethnicity, lncRNA expression level and detected sample types which may influence the heterogeneity. We found that detection and ethnicity unlikely affected the diagnostic accuracy for RCC. By contrast, the lncRNA expression level significantly affected sensitivity. Therefore, the down-regulated lncRNAs may exhibit a higher sensitivity for the diagnosis of RCC. However, we were unable to perform advanced analysis because of the limited number of studies and insufficient number of articles regarding other lncRNAs. Nevertheless, these limited data still fuel our imagination. As we can see, the diagnostic value of single lncRNA was high enough, if we merge some of them, can we improve the diagnostic rate of RCC? Wu *et al.* [35] reported that the AUC of a 5-lncRNA signature, including lncRNA-LET, PVT1, PANDAR, PTENP1 and linc00963 was higher than that of one lncRNA after they detected serum samples from 71 ccRCC patients, 62 healthy controls, and 8 patients with benign renal tumors. Moreover, five-minus-one lncRNA signatures yield that none of the lncRNAs had a higher AUC than the other lncRNAs do [35]. Jicheng Tantai *et al.* [15] reported that combination of lncRNA-XIST and lncRNA HIF1A-AS1 had a higher positive diagnostic efficiency of NSCLC than XIST or HIF1A-AS1 alone. From this point of view, it provided a promising way to determine biomarkers for the diagnosis of RCC. However, further large-scale studies should be conducted to verify this method.

Our study revealed that MALAT1 was correlated not only with clinicopathological features but also with RCC prognosis; MALAT1 was the most investigated lncRNA in RCC. Thus, the application of MALAT1 is possibly the most promising lncRNA for future studies. MALAT1 is a widely expressed and highly conserved nuclear-abundant lncRNA with a length of approximately 8000 nucleotides [37]. MALAT1 was first identified as an independent prognostic biomarker that can predict metastasis and survival in early stage non-small cell lung cancer (NSCLC) [38]. MALAT1 was highly expressed in several cancer types, such as NSCLC [38], gastric cancer [39], colorectal cancer [40], breast cancer [41], cervical cancer [42], prostate cancer [43], nasopharyngeal cancer [44], and renal cancer [25]. The up-regulation of the MALAT1 expression was associated with the clinical parameters and poor prognostic outcome of cancer patients, this finding is consistent with our results. Many molecular mechanisms could explain this relationship. Hirata *et al*. [20] reported that MALAT1 silencing decreased RCC cell proliferation and invasion but increases apoptosis. This phenomenon promoted aggressive RCC through Ezh2 and interacts with miR-205; this phenomenon also regulated EMT and β-catenin signaling pathways in renal cancer cells. Furthermore, miR-125b binded to MALAT1 and decreased the expression level of MALAT1.

Despite the scope of this systematic and comprehensive meta-analysis, several limitations should be considered. First, the number of studies included in our meta-analysis was insufficient and the sample size was limited. Most of these studies contained diverse lncRNAs and used different follow-up endpoints, and one lncRNA was identified by two studies. Hence, a premature result may be obtained. Further studies should be conducted when more eligible studies are published. Furthermore, we did not evaluate publication bias in our study because of inadequate data. As literature-based analyses, studies with positive results were more likely to be published. However, the lack of these analyses can amplify the association between lncRNAs and clinical values of RCC, which may partly affect the interpretation and reliability of results. Second, the cutoff value and method for low or high levels of lncRNA varied in different studies, although qRT-PCR was the standard method used to evaluate the expression of lncRNA, which may cause the heterogeneity of results. Therefore, researchers should develop a cutoff value with enhanced consistency and establish a method to classify high or low lncRNA expression. Finally, a remarkable heterogeneity was observed in the analysis of diagnostic value. The heterogeneity of the subjects was composed of different factors, such as patients' baseline characteristics and different cut-off methods. Although we performed the subgroup analysis and found that lncRNA expression level was one of the sources of heterogeneity, this bias could not be accounted for the entire source.

In summary, our study is the first meta-analysis to evaluate the expression of lncRNAs and clinical values of patients with RCC. Despite these limitations, our analysis revealed that lncRNAs could be considered biomarkers for lymph node metastasis and distant metastasis in early stages. Furthermore, lncRNAs could be potential prognostic markers for RCC. LncRNAs also exhibited high diagnostic accuracy for RCC diagnosis. What's more, MALAT1 was associated with tumor stage and could be considered a biomarker for lymph node metastasis in early stages, and high MALAT1 expression predicted poor survival among patients with RCC. However, further comprehensive, large-scale, and good quality studies should be conducted to confirm our findings and to verify the clinical values of lncRNAs in RCC.

## MATERIALS AND METHODS

### Search strategy

We performed a literature search on up-to-date electronic databases, including Pubmed, Embase, and Web of Science for studies that analyzed the relationship between lncRNAs and clinical values (clinicopathology, prognosis, and diagnosis) in RCC patients on May 25th, 2016. We mainly searched three key aspects “lncRNA”, “cancer”, and “renal”, following was the detail search strategy in Pubmed: *(lncRNA OR lncRNAs OR lincRNA OR lincRNAs OR “long non-coding RNA” OR “long noncoding RNA” OR “long intergenic noncoding RNA” OR “long non protein coding RNA” OR H19)* AND *(cancer OR carcinoma OR neoplasm OR tumor OR tumors OR tumour OR tumours OR malignancy OR metastasis)* AND *(renal OR renals OR kidney OR kidneys OR RCC OR “renal cell carcinoma” OR (renal AND cell AND carcinoma) OR “renal cell cancer” OR (renal AND cell AND cancer)).* The literature covered was limited to human and English. Additionally, we screened the references from many relevant literatures, including all of the identified studies, reviews, and editorials.

### Eligibility criteria and quality assessment

The inclusion criteria were as follows: (1) studies that investigated the association between the expression of lncRNAs and clinicopathological features, and the expression level of lncRNAs had to be divided into two levels: high or low; or (2) studies that detected lncRNA concentrations in serum or tissue and presented sufficient data, including sensitivity, specificity, and sample size, to allow us to perform statistical analysis and construction of two-by-two tables; or (3) studies that investigated the association between lncRNA expression and survival outcome and provided a HR or relative risk (RR), 95% CI or *p* value, and Kaplan-Meier curves or required data obtained by contacting corresponding authors.

The exclusion criteria were as follows: (1) non-English paper; (2) non-human data; (3) studied only in cellular level; (4) letters, case reports, commentaries, conference abstracts or review articles; (5) sample cases fewer than 30; (6) studies focusing on lncRNA genetic alterations, including methylation patterns or polymorphisms; (7) HRs calculated on the basis of multiple lncRNAs; and (8) insufficient data for HR and 95% CI estimation. We included the most recent and informative article when overlapping studies were retrieved.

Two investigators (Jianwen Chen and Yalei Chen) independently assessed the quality of all the included diagnostic studies according to the Quality Assessment of Diagnostic Accuracy Studies-2 (QUADAS-2) criteria [33]. The QUADAS-2 tool comprises four key domains: patient selection, index test, reference standard, flow and timing, and judge bias and applicability. Each domain is assessed in terms of risk of bias, and the first 3 domains are also assessed in terms of concerns regarding applicability. Each item is answered with “yes,” “no,” or “unclear”. The answer of “yes” means low risk of bias, while “no” or “unclear” means the opposite.

### Data extraction

Data were retrieved independently by two investigators (Jianwen Chen and Yalei Chen), and the information of all the included studies were extracted using a predefined sheet which based on the reporting checklists of Preferred Reporting Items for Systematic Reviews and Meta-analysis (PRISMA) [45]. Data retrieved from the articles included the following items: (1) publication information: first author's last name, publication year and study design; (2) patients' characteristic information: study population and regions, sample size, and follow-up duration; (3) RCC cancer information: tumor type, and clinical tumor stage; (4) lncRNA information: detection methods, cut-off definition, and relationship between lncRNAs and survival outcome or clinicopathological features; and (5) sensitivity, specificity, AUC, and sample sizes for diagnostic analysis and two-by-two table construction; (6) HRs, 95 % CI and *p-*value for survival analysis, if available, these data were obtained from the original article; otherwise, corresponding authors were contacted to collect these data; if Kaplan-Meier curves were available, data were extracted from graphical survival plots and HRs were estimated [46].

### Statistical analysis

A test of heterogeneity among studies was conducted using I-squared statistic, I^2^ values of > 50% indicated that there was a substantial between-study heterogeneity existed. The potential sources of heterogeneity were further identified by subgroup analysis. A fixed effect model was applied for the meta-analysis with moderate heterogeneity (I^2^ < 50 %); otherwise, a random effect model was used [47, 48]. A different effect size (ES) was selected for each meta-analysis. (1) OR and a 95% CI were used for the meta-analysis of clinicopathological features. (2) For the prognostic meta-analysis, the ln HR and standard error were used for aggregation of the survival results. An observed HR > 1 implied a worse survival for the group with elevated lncRNA expression. Conversely, an observed HR < 1 implied a worse survival for the group with decreased lncRNA expression [47]. (3) Sensitivity, specificity, PLR, NLR, DOR, SROC curve, and AUC were used for the diagnostic meta-analysis. All analyses were performed using the Stata Statistical software version 12.0 (StataCorp, College Station, TX, USA), and *p* < 0.05 was considered to be significant.
